# *Euphorbia antisyphilitica* Zucc: A Source of Phytochemicals with Potential Applications in Industry

**DOI:** 10.3390/plants10010008

**Published:** 2020-12-23

**Authors:** Romeo Rojas, Julio César Tafolla-Arellano, Guillermo C. G. Martínez-Ávila

**Affiliations:** 1 Laboratory of Chemistry and Biochemistry, School of Agronomy, Autonomous University of Nuevo Leon, General Escobedo, Nuevo Leon 66050, Mexico; romeo.rojasmln@uanl.edu.mx; 2 Basic Sciences Department, Laboratory of Biotechnology and Molecular Biology, Antonio Narro Agrarian Autonomous University, Saltillo, Coahuila 25315, Mexico; jtafare@uaaan.edu.mx

**Keywords:** candelilla plant, antioxidant properties, antimicrobial activity, polyphenols, candelilla cultivation, candelilla wax

## Abstract

*Euphorbia antisyphilitica* Zucc, better known as the candelilla plant, is one of the 10 non-timber forest products of greatest economic importance in the desert and semi-desert regions of Mexico. Moreover, it is a potential source of some functional phytochemicals such as polyphenolic compounds, wax and fiber, with potential applications in food, cosmetic and pharmaceutical industries. Thus, this review aims to describe these phytochemicals and their functional properties as antimicrobial, antioxidant, reinforcing and barrier agents. In addition, a suitable valorization of the candelilla plant and its byproducts is mandatory in order to avoid negative effects on the environment. This review provides, for the first time, an overview of the alternative methodologies for improving candelilla plant production, pointing out some of the agricultural aspects of the cultivation of this plant.

## 1. Introduction

**Figure 1:**
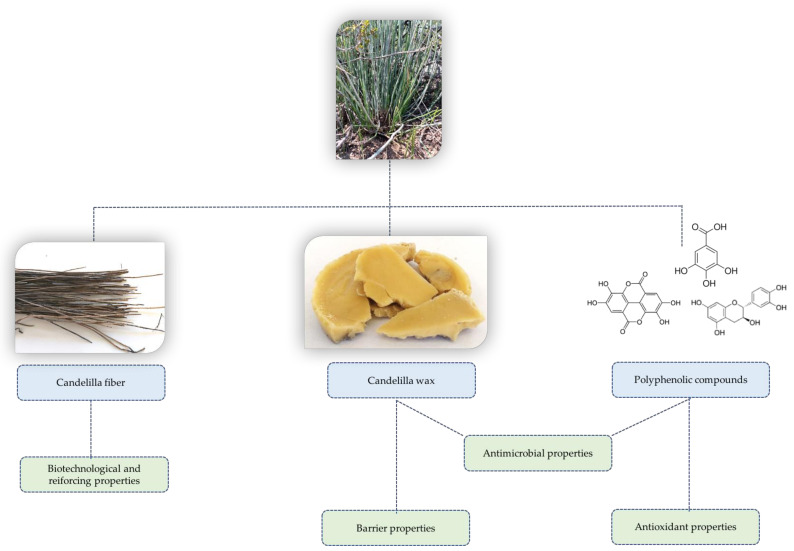
Phytochemicals and functional properties of E. antisyphilitica Zucc.

The genus *Euphorbia* belongs to the *Euphorbiaceae* family, and it is one of the largest genera of higher plants, with more than 2000 recognized species, such as *Euphorbia thymifolia*, *Euphorbia neriifolia*, *Euphorbia antiquorim*, *Euphorbia chamaesyce*, *Euphorbia helioscopia* and *Euphorbia antisyphilitica* [1,2]. One of the most important species of this family is *E. antisyphilitica* Zucc, better known as the candelilla plant, which is used traditionally as an herbal remedy in countries such as India and other arid and semiarid countries [1]. It naturally grows in the desert and semi-desert regions of northern Mexico, and this non-timber plant is a very important economic resource for the people living in these areas, due to the extreme climatic conditions which restrict agricultural activities [3,4]. The candelilla plant grows in clusters, with thin wax-covered stems that protect them as thick layers giving tolerance against environmental conditions (i.e., temperature variations) and biotic agents (i.e., insects) [5,6]. Therefore, this plant is mainly used to obtain wax, which can be considered as a multipurpose agent for several industries due to its unique properties and multiple applications in the formulation of food, cosmetic and pharmaceutical products. In addition, the candelilla plant has proven to be a good source of other useful phytochemicals such as fiber, wax and polyphenolic compounds (i.e., catechin and ellagic acid), as shown in Figure 1. In this sense, this review provides interesting information generated from around the world about some constituents and bioactive compounds from the candelilla plant and their functional properties. 

## 2. Phytochemicals Present in Candelilla Plant and Byproducts

**Figure 2:**
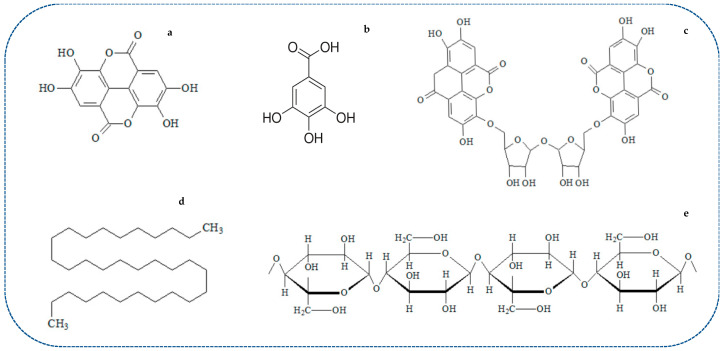
Main phytochemicals identified in E. antisyphilitica and byproducts: (**a**) ellagic acid; (**b**) gallic acid (candelilla whole plant); (**c**) candelitannin (candelilla byproducts); (**d**) hentriacontane (wax); (**e**) cellulose (candelilla bagasse fiber).

Usually, a proximal chemical analysis is carried out to determine the chemical constituents of a plant material. In this sense, the chemical analysis of candelilla plants has been reported by Rojas-Molina et al. [5] and Ventura-Sobrevilla et al. [7], as shown in Table 1. Although the chemical composition of plants is strongly influenced by factors such as weather conditions, and season and genetic variability, it can be seen from the reported proximal analysis that lipids and ashes are the major constituents of candelilla plants, which are related to the presence of candelilla wax as mentioned above. Moreover, it has been reported that the candelilla plant possesses a large number of high-quality bioactive phytomolecules with potential applications in different industries acting as antimicrobial and antioxidant agents, and providing other technological advantages [8,9,10,11,12,13]. Figure 2 shows the structure of the main phytochemicals identified in the candelilla plant and its byproducts.

Table 2 shows the phytochemicals obtained from the candelilla plant and its byproducts, and the functional activity of these compounds. Since candelilla wax is one of the most important constituents of E. antisyphilitica, it is necessary to know its chemical composition, which has been reported as follows: n-alkanes (hentriacontane as the main component) > high molecular weight esters > alcohols and sterols > free acids (7–9%) [4,5,14,15,16,17]. Another important structural component of the candelilla plant is fiber, which can be used as a support in the production of hydrolytic enzymes and as a reinforcing agent, as explained below [11,18]. It has been established that candelilla bagasse fiber (CBF) comprises cellulose (45%), hemicellulose (16%), lignin (37%), pectin (1.8%), wax (0.5%) and water-soluble extract (8–12%), a composition that is similar to other natural fibers (i.e., sisal and jute fiber) with industrial applications [11]. In addition, the presence of some polyphenolic compounds has also been reported in the candelilla plant. Rojas-Molina et al. [5] and Ventura-Sobrevilla et al. [7] detected the presence of hydrolysable and condensed tannins, as well as the presence of catechin, ellagic and gallic acid. The first study about the extraction of ellagic acid from E. antisyphilitica was reported by Aguilera-Carbo et al. [19]; the authors reported that the candelilla plant has at least double the quantity of this phenolic compound than other plant materials such as Turnera diffusa and Jatropha dioica. Recently, the putative structure of a high molecular weight (860.7 g mol^−1^) ellagitannin called candelitannin has been reported in candelilla residues [9]. Finally, flavonoids and other molecules (samonins and quinons) can be found in the methanolic extracts of the candelilla plant [8]; however, no further information has been provided about these components. Regardless of these reports, there is currently limited information on the chemical characterization of this plant, which provides an opportunity for innovative studies in this field due to its economic importance.

## 3. Agricultural Aspects of Cultivation

According to a very recent study published by Vargas-Pineda et al. [20], candelilla plants have a distribution area of more than 19.1 million hectares in North America under the current climatic conditions. Nevertheless, although the collection of candelilla plants is an important economic activity for communities from northern Mexico, any overharvesting should be avoided. Villa-Castorena et al. [21] conducted a comprehensive study related to the production of candelilla seedlings by cuttings. In this study, four eco-types of candelilla plants called Cuencamé, Cuatrociénegas, Tlahualilo and Viesca, four growing substrates (sandy soil, mixture of river sand and coconut fiber (1:1), mixture of river sand and peat moss (1:1) and mixture of peat moss, perlite and vermiculite (1:1:1)), and four chemical treatments (ProRoot, magic root, phenoxyiacetic acid and a treatment without chemical application) for root promotion were evaluated. According to the authors, untreated Cuatrociénegas eco-type had the highest ability to promote superior rooting of the cuttings due to its special genetic characteristics which allow this eco-type to produce more endogenous auxins for emitting more roots without the presence of other chemicals. For the other eco-types, the cuttings treated with growth media of peat moss with perlite and vermiculite and the mixture of river sand and peat most improved the percentage of rooted cuttings and the shoot growth of this plant. In this sense, the production of candelilla plants can be explored in controlled environments such as greenhouses in order to provide seedlings of good quality for the reforestation of affected areas. In addition, it has been reported that candelilla plants exhibited greater relative growth rate (0.15 g·g^−1^·d^−1^) and relative water content (88%), which was associated with the physiological status of this plant, allowing its consideration for suitable growth and production as green roofs in arid regions [22].

On the other hand, some stress conditions in the cultivation of candelilla plants, such as the use of lime on the cultivation soil, can improve the production of secondary metabolites (i.e., epicular wax) due to an increase in the pH of the plant tissues due to the alkaline conditions in the soil [23]. According to the authors, candelilla plants cultivated on a liming soil (10 g per pot) reach, on average, more than 54% extractable wax when compared to the control and other abiotic stress conditions (plastic cover and solar reflection). However, no additional studies related to the effect of the stress conditions on additional secondary metabolite expression of this plant were found, which enables the development of new research in this regard.

## 4. Functional Properties of the Phytochemicals

Due to their functional characteristics, phytochemicals of candelilla plant have the potential to be used in several processes in food, cosmetic and biotechnological industries, as they have demonstrated antimicrobial and antioxidant properties (phenolics and wax), good barrier properties (wax) and reinforcing and biotechnological properties (fiber).

### 4.1. Antimicrobial Properties

In the last decade, interest in the study of the phytochemical constituents of E. antisyphilitica has grown (Table 2). In addition, it has been reported that plants from northern Mexico have several secondary metabolites to which some biological activities are attributed [8,12]. Thus, the studied molecules have proven to be highly effective at acting as antifungal and antimicrobial bacterial agents [8,9,12]. In their study, Serrano-Gallardo et al. [12] evaluated non-toxic methanolic extracts of four different plants used as a traditional remedy from a semi-desert region in Mexico, including E. antisyphilitica. In their study, the authors evaluated different concentrations of the plant extracts (500, 100 and 2000 μg mL^−1^) against two reference bacterial strains, Staphylococcus aureus BAA44 and Klebsiella pneumoniae 9180, and four bacteria isolated from clinical samples (S. aureus, K. pneumoniae, Pseudomonas aeruginosa and E. coli). From the obtained results, the authors found that the candelilla extracts presented antimicrobial activity against all the tested bacteria at 500 μg mL^−1^ (as a minimum inhibitory concentration), which can be related to the phytochemical profile detected in this plant (saponins and quinones). This is according to the previous results reported by Vega-Menchaca et al. [8], who determined that methanolic extracts from candelilla leaves showed antimicrobial activity against clinically isolated bacteria strains such as S. aureus, E. coli O157 and Enterobacter aerogenes 9183. In comparison, in the study of Vega-Menchaca et al. [8], the minimum inhibitory concentration needed for the inhibition of S. aureus was lower (26.8 μg mL^−1^) than that reported by Serrano-Gallardo et al. [12], which could be due to the presence of flavonoids in candelilla extracts evaluated by Vega-Menchaca et al. [8], as explained by the authors. However, it is important to consider other factors which can affect phytochemical composition of candelilla plants such as weather conditions and season. 

Candelilla byproducts also contain other bioactive phenolic compounds that have been related to the antimicrobial activities of candelilla extracts against Erwinia amylovora, Xanthomonas axonopodis and Clavibacter michiganensis [10]. According to the authors, polyphenolic compounds from the hydro-alcoholic extracts obtained from the candelilla byproducts are responsible for the inhibitory effect against the pathogenic bacteria. This antimicrobial potential could be related to the presence of ellagitannins such as candelitannin in these plant materials which has shown effective antifungal properties against four phytopathogenic fungal strains: Alternaria alternata, Fusarium oxysporum, Colletotrichum gloeosporioides and Rhizoctonia solani [9]. The antimicrobial activities exhibited by the phenolic-rich extracts from candelilla plants may be attributed to the interaction of these compounds with the cell membrane causing several modifications to it and changes in various intracellular functions, such as interspecific permeability causing microbial death [24]. Thus, polyphenolic compounds from the candelilla plant can be an important alternative to traditional antimicrobial agents, and a complement to antibiotic therapy. On the other hand, it has been proven and hypothesized that, by itself, candelilla wax has antimicrobial effects on E. coli ATCC 10536 and some fungal strains such as Botrytis cinerea, Colletotrichum gloeosporioides and Fusarim oxysporum, when it is used in the formulation of edible coatings and films [25,26]. However, due to scarce information in the literature, the specific role of candelilla wax in this effect is still unknown.

### 4.2. Antioxidant Properties

Free-radical scavenging of extracts from candelilla byproducts was evaluated by Burboa et al. [10]. The authors reported that ethanolic extracts can inhibit more than 88% of the DPPH^•^ radicals according to the methodology used for this analysis, which was related to the presence of phenolic compounds in these extracts. This is in line with the findings reported for other species of the Euphorbiaceae family, in which polyphenolic compounds have been detected, such as tannins and flavonoids, which are well known for their antioxidant capacity [1]. Nevertheless, regardless of the high content of phenolic compounds, the evaluation and exploitation of antioxidant properties of candelilla plant extracts are still scarcely explored. In this sense, more research should be conducted with the purpose of generating new and innovative knowledge about the antioxidant properties of the polyphenolic compounds and other phytochemicals from this plant material, as they have great potential to be applied in food, pharmaceutical and cosmetic industries.

### 4.3. Barrier Properties of Candelilla Wax

Barrier properties of candelilla wax against water vapor and gas transfer are two of the most important features for researchers, and they are usually measured by the formulation of edible coatings and films. The moisture barrier property is the most recognized characteristic of candelilla wax, which is based on its hydrophobic nature and capacity to form a compact network in synergy with other structural compounds such as proteins and carbohydrates [6,14,27,28]. According to Kowalczyk et al. [27], candelilla wax-based films exhibit low water and oxygen permeability compared to other lipid sources, which decreases when the concentration increases (from 0.5 to 2.0%). This can be attributed to its ability to increase film surface hydrophobicity. In the same way, some authors have recorded a decrease in the weight loss of food products such as avocado, “Golden Delicious” apples, Fuji apples and Persian limes. This could be due to the morphology of the coating surface which has a homogenous particle size and less roughness, reducing the open area of the emulsified solids network and therefore avoiding loss of moisture from the fruit [6,28,29,30]. However, more studies must be conducted regarding the low oxygen permeability of candelilla wax-based coating and films, as it can affect some quality and sensory attributes of coated food products due to the anaerobic respiration and changes in pH [31,32].

### 4.4. Other Functional Applications

Due to the candelilla wax extraction process generating large amounts of lignocellulosic waste, they can be used for different technological purposes. Regarding the biotechnological aspects, candelilla fiber has been used as a support for fungal growth to the production of ellagitannase due to its ellagitannin content [18]. In this study, the authors evaluated four agroindustrial byproducts, and, although it was concluded that candelilla stalks have great potential for use as support for solid-state fermentation (SSF), the lowest values of ellagitannase were obtained using this plant material. Thus, future studies must focus on the standardization of this biotechnological process in order to increase enzyme-activity titles. On the other hand, due to their physico-chemical characteristics, candelilla fiber has also been used as a reinforcing agent in the formulation of new composites based on polypropylene (PP) and CBF [11]. In this study, the authors found that this byproduct is stable at around 200 ºC, which improves the thermal stability and tensile properties of the CBF–PP composites due to the crystallinity index of the cellulose and the disruption of the free moment of the polymeric chains in the matrix, respectively. In a more recent study, it was demonstrated that candelilla fiber contains intracuticular wax and resins, which has the benefit of being a compatibilizer between the fiber and the polypropylene [35]. This is according to a recent study by Pulido-Barragán et al. [34], who reported that CBF is a good plant material for obtaining cellulose nanocrystals, which can be used as a reinforcing, structural or thermal agent, as well as for 3D printing and as a constructive nanocellulosic paper agent, among other technical applications due its the specific physico-chemical properties. Although these studies were well conducted, limited information on the application of candelilla fiber is available, providing an opportunity for the investigation of other functional properties of this plant material.

## 5. Final Remarks and Perspectives

Since the candelilla plant is an endemic of the semiarid regions of northern Mexico, this country has been recognized for its potential to be the main producer of candelilla wax. However, this review provides interesting and innovative information associated with the promising applications and sustainable valorization of other phytochemicals from the candelilla plant not published elsewhere. In addition, it provides an opportunity for developing more investigations into the physicochemical characterization of polyphenolic compounds (different to ellagic acid), and the fiber present in the candelilla plant and its byproducts. Furthermore, considering its great potential to be used as a source of components with several functional applications, researchers should focus more on the evaluation, stability and application of the phytochemicals of this plant, as they could help to replace synthetic molecules used at the industrial level. In addition, the waste disposal for animal feed (after the SSF process) and the evaluation of CBF as a reinforcing agent (after the extraction of wax and phenolic compounds) could be of great interest for creating a “zero waste” process as an integral use of this plant resource.

## Figures and Tables

**Table 1:** Proximal chemical analysis carried out in candelilla plants.

	g per 100 g of Dry Material	
Component	[5]	[7]
Moisture	0.4 ± 0.0006	4.44
Total solids	99.6 ± 1.81	95.6
Lipids	15.9 ± 1.119	15.92
Crude fibers	9.0 ± 1.217	9.05
Proteins	2.3 ± 0.0571	1.42
Ashes	10.9 ± 0.315	10.89
Total sugars	0.27 ± 0.043	23.93
Reducing sugars	0.16 ± 0.021	

**Table 2:** Phytochemicals from candelilla plant, methods for characterization, registered yields and potential functional activity.

Chemical Nature	Phytochemicals	Yield	Analysis Method for Chemical Characterization	Potential Activity	Reference
Phenolic compounds	Ellagic acid	2.18 ± 0.39 mg g^−1^	HPLC	Anti-atherosclerotic, antimicrobial and antioxidant activity	[19]
	Hydrolysable tannins	0.56 ± 0.010	Colorimetric methods	Antioxidant activity	[5]
	Condensed tannins	0.16 ± 0.013			
	Ellagic acid	2.2 ± 0.15 mg g^−1^	HPLC		
	Gallic acid	0.6 ± 0.03 mg g^−1^			
	Catechin	0.2 ± 0.02 mg g^−1^			
	Ellagic acid	6.06−7.09 mg g^−1^		Antitumoral, antiviral, antioxidant activity	[33]
	The presence of ellagitannins and ellagic acid was hypothesized	NI	NI	Antimicrobial, antioxidant and antihemolytic activities	[10]
	Candelitannin		FT-IR and HPLC	Antifungal activity against phytopathogenic agents	[9]
Carbohydrates	Fiber		Water absorption index and critical humidity point and SEM	Great potential to be used as support in solid-state fermentation process	[18]
			FT-IR, thermogravimetry and X-ray diffraction	As a reinforcing agent for polypropylene composites	[11]
			FT-IR, thermogravimetry, X-ray diffraction and SEM	Reinforcing, structural and thermal agents	[34]
			Dynamic mechanical analysis and SEM	Improve the mechanical properties of polypropylene composites	[35]
Lipids	Candelilla wax		NI (No identified)	Excipients clinical applications	[36]
				Reduces water permeability of edible films	[15]
				In the encapsulation of fertilizers with slow-release properties	[13]
			HPLC and GC	Structural agent in food systems	[16]
					[17]
			NI	Antimicrobial and water resistance properties	[25]
		2–5 g 100 g^−1^		Can be used to obtain petroleum	[37]
Other compounds	Saponins and quinones	NI	NI	Antimicrobial potential	[12]

SEM = scanning electron microscope; FT-IR = Fourier transform infrared spectroscopy; HPLC = high-performance liquid chromatography; GC = gas chromatography.
